# The fungal blind spot: Why marine carbon models ignore a key player

**DOI:** 10.1371/journal.pbio.3003840

**Published:** 2026-06-17

**Authors:** Marlis Reich

**Affiliations:** University of Bremen, MARUM, FB2, Bremen, Germany

## Abstract

Marine fungi were assumed to have a minor role in the carbon cycling, unable to compete with bacteria. A new PLOS Biology study challenges this dogma, showing fungi can dominate labile dissolved organic matter assimilation, reshaping our understanding of ocean carbon retention and storage.

## Teaser

Marine fungi were assumed to have a minor role in carbon cycling, unable to compete with bacteria. A new PLOS Biology study challenges this dogma, showing fungi can dominate labile dissolved organic matter assimilation, reshaping our understanding of ocean carbon retention and storage.

For decades, our understanding of heterotrophic microbial diversity, functionality, and carbon cycling in the oceans and transition zones has been dominated by a focus on prokaryotes. Marine fungi have been systematically neglected because the terrestrial picture of fungi does not translate directly to marine habitats. In the ocean, fungal structures are microscopic and thus difficult to detect. They occur as unicellular organisms with a flagellum (zoospores), yeasts, or multicellular structures with thin, branched filaments (hyphae). Only in the last two decades have fungi become a focus of marine microbial ecology. Today, we know fungi are ubiquitous in the ocean, exhibiting vast taxonomic and ecological diversity, such as saprotrophs decomposing dead organic matter [1]. However, a new study of Trejos-Espeleta and colleagues [2] now challenges this historical neglect by demonstrating fungal dominance in the assimilation of dissolved organic matter.

Nevertheless, to what extent marine saprotrophic fungi have adapted to marine environments remains strikingly poorly understood. The fungal lineage evolved in an aquatic environment, where the earliest fungi were parasitic unicellular organisms. The diversification of fungi and the development of complex hyphae occurred later, after fungi colonized land. As the evolutionary lineage of plants, the Streptophyta, diversified and shaped their ecosystems, fungi evolved into the Dikarya [3]. It is assumed that these highly evolved fungi returned to the ocean in several secondary transitional steps [4]. This raises critical questions: Can higher saprotrophic fungi decompose marine-derived organic matter? Where might they find a competitive niche to outcompete smaller and more abundant bacteria with shorter generation times for organic material?

Given their cell size and architecture and broad enzyme capabilities, marine saprotrophic fungi of the Dikarya were assumed to predominantly exploit more complex and particulate organic matter. In contrast, easily degradable dissolved organic matter was considered primarily the niche of bacteria. The new study by Trejos-Espeleta and colleagues [2] shatters this paradigm: fungi in microbial communities are not just competitors but can outperform bacteria in dissolved organic matter assimilation, demonstrating that carbon cycling in marine ecosystems is far more complex than previously assumed and urgently needs to be studied with a holistic angle. Using quantitative stable isotope probing, Trejos-Espeleta and colleagues [2] show that marine fungi in an Arctic fjord efficiently assimilate labeled dissolved organic matter, primarily in sediments, but also in seawater. At times, they exhibit higher metabolic efficiency than prokaryotes in sediments.

**Fig 1:**
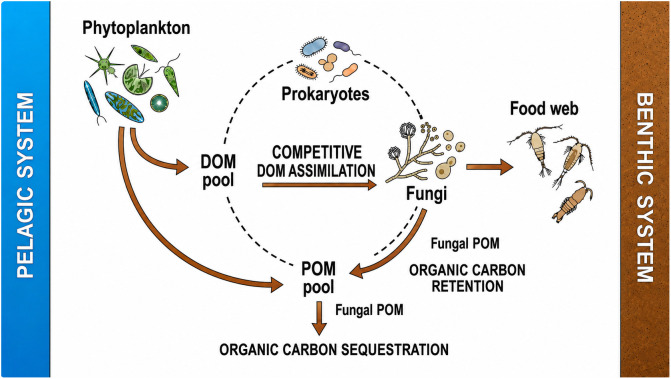
Conceptual model of fungal contributions to the marine carbon cycle in pelagic and benthic systems, integrating findings from the new study by Trejos-Espeleta and colleagues. Marine fungi are part of the microbial loop, where organic material (e.g., from phytoplankton) is degraded and transformed. Organic matter occurs in dissolved or particulate forms. Trejos-Espeleta and colleagues [2] demonstrated that fungi can competitively assimilate dissolved organic matter (DOM) to produce biomass. Fungal biomass itself contributes to the pool of particulate organic matter (POM), thereby retaining carbon within the system. When it sinks or is buried in deeper sediment layers, fungal particulate organic matter may also enhance long-term carbon sequestration. Figure created using ChatGPT based on the author’s detailed prompt and key terms.

The paper also tackles a glaring critical gap: the mechanisms of ocean carbon retention, where fungi have been entirely overlooked in existing models. Here, the work by Trejos-Espeleta and colleagues [2] is equally groundbreaking: their findings show that efficient fungal metabolism converts labile dissolved organic matter into stable biomass, retaining the carbon in the system (Fig 1).

Fungi can rapidly accumulate substantial biomass, even in marine environments. How must we rethink carbon retention and sequestration if part of the estimated 0.32 gigatons of carbon of marine pelagic fungal biomass stems from dissolved organic matter assimilation? This estimate derives solely from counts of unicellular fungi (2–6 µm), which comprised 99% of observations during an Atlantic transit cruise. Yet, even these unicellular fungi already contribute to the marine organic particle fraction, which can exceed 1% of total particulate organic matter in the water column [5].

But what about sediments, where hyphae-forming fungi dominate and their hyphal networks span scales >50 µm [6]? How large is the fungal biomass there, and how much dissolved organic matter is incorporated into particles of fungal biomass? At present, however, we lack the data to quantify this. The high carbon retention via fungal biomass should not be equated with sequestration, though. There are too many unknowns, such as how much fungal biomass enters the food web and is respired, and how effectively other microbes decompose it.

The paper by Trejos-Espeleta and colleagues [2] shows that fungi assimilate more dissolved organic matter than bacteria, accompanied by higher biomass, but in a subset of samples. However, this is the second study from William Orsi’s lab to report sporadic fungal dominance in carbon assimilation across marine microbial communities [7]. Future studies must employ higher temporal and spatial resolution, targeting gradients such as carbon concentration, the water–sediment interface, and oxic–anoxic transitions. Moreover, we must prioritize understanding the mechanisms of fungal dissolved organic matter decomposition and the molecular basis of their competitive carbon assimilation within microbial communities. The study by Trejos-Espeleta and colleagues [2] raises critical questions about the ecological roles of marine fungi in the carbon cycle from decomposition and transformation to retention and sequestration. It lays the foundation for new hypotheses and compels even skeptics of fungal involvement in marine carbon cycling to reconsider their stance.
